# Biofunctionalization of Porous Titanium Oxide through Amino Acid Coupling for Biomaterial Design

**DOI:** 10.3390/ma16020784

**Published:** 2023-01-13

**Authors:** Paolo Canepa, Danijela Gregurec, Nara Liessi, Silvia Maria Cristina Rotondi, Sergio Enrique Moya, Enrico Millo, Maurizio Canepa, Ornella Cavalleri

**Affiliations:** 1 Dipartimento di Fisica and OPTMATLAB, Università di Genova, Via Dodecaneso 33, 16146 Genova, Italy; 2 Department of Chemistry and Pharmacy, Chair of Aroma and Smell Research, Friedrich-Alexander-Universität Erlangen-Nürnberg, Schlossplatz 4, 91054 Erlangen, Germany; 3 Dipartimento di Medicina Sperimentale, Università di Genova, Viale Benedetto XV 1, 16132 Genova, Italy; 4 Soft Matter Nanotechnology Group, CIC biomaGUNE, Basque Research and Technology Alliance (BRTA), Paseo Miramón 182, 20014 San Sebastián, Spain; 5 INFN, Sezione di Genova, Via Dodecaneso 33, 16146 Genova, Italy

**Keywords:** porous titanium oxide, aminophosphonates, amino acid coupling, Fmoc-Leucine, nanoshaving, AFM, XPS

## Abstract

Porous transition metal oxides are widely studied as biocompatible materials for the development of prosthetic implants. Resurfacing the oxide to improve the antibacterial properties of the material is still an open issue, as infections remain a major cause of implant failure. We investigated the functionalization of porous titanium oxide obtained by anodic oxidation with amino acids (Leucine) as a first step to couple antimicrobial peptides to the oxide surface. We adopted a two-step molecular deposition process as follows: self-assembly of aminophosphonates to titanium oxide followed by covalent coupling of Fmoc-Leucine to aminophosphonates. Molecular deposition was investigated step-by-step by Atomic Force Microscopy (AFM) and X-ray Photoemission Spectroscopy (XPS). Since the inherent high roughness of porous titanium hampers the analysis of molecular orientation on the surface, we resorted to parallel experiments on flat titanium oxide thin films. AFM nanoshaving experiments on aminophosphonates deposited on flat TiO_2_ indicate the formation of an aminophosphonate monolayer while angle-resolved XPS analysis gives evidence of the formation of an oriented monolayer exposing the amine groups. The availability of the amine groups at the outer interface of the monolayer was confirmed on both flat and porous substrates by the following successful coupling with Fmoc-Leucine, as indicated by high-resolution XPS analysis.

## 1. Introduction

Transition metals have been extensively studied in recent decades for the development of orthopedic prosthetic materials. The native oxide layer that spontaneously forms on their surface upon exposure to the environment helps reduce the release of metal ions into the host tissue, promoting the biocompatibility of the material and reducing the risk of metallosis. Over the years, numerous studies have been conducted to optimize the properties of the surface oxide layer to promote implant osseointegration. Anodic oxidation is a widely used method for this purpose because it allows, through appropriate choice of anodizing conditions, to improve the passivation of the metal by growing thick oxide films enriched in osteoconductive elements, such as Ca and P, with a highly porous structure, which favors osseointegration [1,2,3,4,5,6].

Along with osseointegration, a critical aspect for successful prosthetic implantation is to impart anti-infective properties to the implant surface in order to reduce bacterial adhesion and proliferation. In fact, the risk of infection is a major cause of implant failure [7]. According to the ‘race for the surface’ concept, the presence of a foreign body sparks a competition between bacteria and host cells to colonize the surface of the implant [8]. If the race is won by the host cells, the surface will be covered by the cells and will be less vulnerable to bacterial colonization. On the other hand, if bacteria win the race, the implant surface will eventually be covered by biofilms, and the host cells will be hampered by bacterial virulence factors, ultimately leading to infection.

This has motivated numerous studies focused on modifying the surface of implants to develop biomaterials with antibacterial properties. Various alternative approaches have been proposed, ranging from modifying the surface morphology of the material to altering the chemical properties of the surface by depositing antibacterial coatings [9].

Several methods have been explored to tune the nanoscale surface structure to prevent bacterial colonization [10]. Among others, high aspect ratio nanostructured surfaces obtained by hydrothermal treatment [11] or via reactive ion etching [12] have been shown to reduce bacterial biofilm formation effectively.

Along with surface morphology, great attention has been paid to modifying the chemical properties of the surface to improve its antibacterial properties. In this respect, surface coatings with hydrophilic, highly hydrated uncharged polymers, e.g., polyethylene-glycol [13,14] or zwitterionic films [15,16] have been widely investigated to confer low adhesion, antifouling properties to the surface. In addition to low-adhesion surfaces, intrinsically bioactive antimicrobial coatings, including coatings involving quaternary ammonium salts [17], polymeric materials, such as chitosan [18] and its potentiated derivatives, and several metals [19], such as silver, zinc and copper have been widely investigated.

Antimicrobial peptides are another class of molecules that are currently receiving large attention as promising compounds to treat antibiotic resistant bacterial infections because of their inhibitory effects against bacteria, fungi, parasites, and viruses [20]. The emergence of antibiotic-resistant microorganisms and the increasing concerns about the use of antibiotics prompted the development of this class of antimicrobial molecules.

A critical point when developing new antibacterial coatings concerns the way the coating is applied and stabilized on the biomaterial to be resurfaced. Current approaches to achieving functional antifouling/antibacterial coatings rely on different strategies, from simple dipping [21], to polymer grafting [22] up to deposition of self-assembled mono- or multilayers [23]. Molecular self-assembly is a very versatile strategy to anchor functional biomolecules to inorganic surfaces [24,25,26].

Here, as a first step toward the ultimate goal of covalently binding antimicrobial peptides to porous titanium oxide surfaces, we focus on the covalent coupling of a single amino acid, as representative constitutive unit of antimicrobial peptides, to porous titanium oxide through the self-assembly of aminophosphonates as the coupling agent. 

Phosphonate self-assembly on oxide surfaces has been widely explored [25,27] as a method to produce functional interfaces with a large range of applications, from semiconductor nanotechnology [28] to biomaterial development [29]. The self-assembly of poly(ethylene glycol) functionalized alkane phosphates on flat titanium oxide has been investigated to reduce nonspecific protein adsorption [30]. Diphosphonic acids self-assembled on native titanium oxide were treated with zirconium tetra(tert-butoxide) to give surface Zr complex species suitable for further coupling with cell adhesive RGD peptide [31].

Here we use amino-terminated phosphonates to obtain amino-terminated interfaces exploitable for the covalent binding of amino acids through the formation of peptide bonds. We present a step-by-step characterization of the molecular functionalization of the titanium oxide surface obtained by coupling Atomic Force Microscopy (AFM) and X-ray Photoemission Spectroscopy (XPS). Our target surface is porous titanium oxide obtained by anodic oxidation. Since the high surface roughness of anodized titanium, a sought-after feature for osseointegration purposes, hampers a detailed characterization of the molecular orientation on the surface, a parallel characterization has been conducted on flat titanium oxide films to obtain additional information on phosphonate self-assembly and molecular orientation. The formation of an oriented aminophosphonate self-assembled monolayer exposing the amino group is indeed a key aspect for the subsequent amino acid anchoring to the surface.

## 2. Materials and Methods

### 2.1. Materials

#### 2.1.1. TiO_2_ Porous Substrates

Titanium foil 99.6+% purity (Advent Research Materials Ltd., Eynsham, UK) was mechanically cut to 1 cm × 1 cm squares and polished using a Struers Labopol-5 machine with grinding plane rotating at 200 rpm (SiC papers with P1000 ÷ P2500 grain size of Fepa-P scale). After mechanical polishing, samples were ultrasonically rinsed for 5 min in Milli-Q water, 5 min in ethanol, 20 min in acetone, rinsed again in Milli-Q water, and finally dried in a N_2_ flow.

Mechanically polished samples were anodized in 1 M sulphuric acid (H_2_SO_4_, 95%, Honeywell Fluka^TM^, Charlotte, NC, USA) aqueous solution, using a two-electrode cell with a Pt grid as counter-electrode. An Agilent N5751A (Keysight Technologies Inc., Santa Rosa, CA, USA) was used as power supply. A LabVIEW (National Instruments) procedure was used to control potential and current during anodizing. Samples were immersed in the electrochemical cell right before beginning anodizing, and they were withdrawn from solution and sonicated in Milli-Q water as soon as the process ended. The solution was stirred during the entire process. Samples were anodized for 15 s in galvanostatic regime with a current value of 2 A using a limiting potential of 200 V.

#### 2.1.2. TiO_2_ Flat Substrates

TiO_2_ thin films were prepared on glass coverslips by direct current (dc) magnetron reactive sputtering in an ATC 1800 UHV sputtering system (AJA International Inc., Scituate, MA, USA) equipped with a load-lock transfer chamber. The base pressure in the chamber was kept at ∼1.2 × 10^−8^ Pa. Glass slides were first chemically cleaned and then after being placed in the sputtering chamber they were further cleaned in argon plasma for 3 min. For coating, a 2 in. diameter Ti target (99.99% purity, AJA International Inc., Scituate, MA, USA) was used, with a power of 228 W, in the argon/oxygen atmosphere generated by combining 10 sccm of argon flow with 20 sccm of oxygen flow at 0.4 Pa working pressure. Substrate-to-target distance was kept at 4 cm, while rotating the substrates at 80 rpm. The sputtering was performed at room temperature for 120 min, resulting in transparent TiO_2_ films of ∼30 nm thickness. Film thickness was calculated as described in [32].

#### 2.1.3. Reagents

We purchased 12-aminododecylphosphonic acid hydrochloride salt ((NH_2_)−(CH_2_)_12_−PO(OH)_2_ HCl, 95% pure) from SiKÉMIA (34790 Grabels, France) and used it without further purification.

Fmoc-Leucine (Fmoc-Leu-OH) (see SI for structural formula) was purchased from Advanced Biotech (20822 Seveso, Italy) and used without further purification.

N,N-diisopropilethylamine (DIPEA, Sigma-Aldrich-Merck KGaA, Darmstadt, Germany), 1-Ethyl-3-[3-dimethylaminopropyl]carbodiimide hydrochloride (EDC, 98.0%, Honeywell Fluka^TM^, Charlotte, NC, USA), absolute ethanol (99.8%, Sigma-Aldrich-Merck KGaA, Darmstadt, Germany), and acetone (analytical grade, Fisher Chemicals, 20053 Rodano, Italy) were used as received. Ultrapure Milli-Q water (Millipore-Merck KGaA, Darmstadt, Germany) with resistivity ≥ 18 MΩ·cm was used.

#### 2.1.4. Aminophosphonate Deposition and Leucine Binding

TiO_2_ samples were treated with oxygen plasma before molecular deposition (15 min @ 200 W power with 15 sccm of oxygen flow) and subsequently quenched in Milli-Q water. Following the protocol adopted in previous work [33], aminophosphonate deposition on TiO_2_ was carried out in 0.2 mg/mL ethanolic solutions at 60 °C for 24 h. We note that a heating step, either during self-assembly or as post-deposition annealing, is usually applied to favor phosphonate coupling to oxide surfaces [28]. After molecular deposition, samples were rinsed in absolute ethanol, dried under a N_2_ stream and analyzed by AFM and XPS.

After aminophosphonate deposition, samples intended for coupling with amino acids were immersed for 4 h in a 0.6 mg/mL Fmoc-Leucine solution in Milli-Q water with EDC (1 eq) and DIPEA (1 eq), used to activate the carboxylic group. The fluorenylmethoxycarbonyl (Fmoc) protection on the α amino group of Leucine was used to avoid coupling reactions between the carboxyl and amino group of amino acid. In fact, to create the bond between the amino group of aminophosphonate and the free carboxyl group of Fmoc-Leucine an activating reactive carbodiimide such EDC was used. This compound promotes the activation of carboxyl groups and subsequent nucleophilic attack by primary amines. First, EDC activates the carboxyl group of Fmoc-Leucine and forms an amine reactive O-acylisourea intermediate that spontaneously reacts with primary amine to form an amide bond and an isourea by-product. After this conjugation, the Fmoc protecting group can be removed by rinsing with a piperidine solution in N,N-dimethylformamide. In the present study, Fmoc was not removed since the XPS signal of the Fmoc aliphatic oxygen was used as an additional fingerprint of Fmoc-Leucine binding.

### 2.2. Methods

#### 2.2.1. Atomic Force Microscope

AFM experiments were carried out using a JPK NanoWizard IV microscope (Bruker, Billerica, MA, USA). Tapping mode AFM imaging was performed using Si cantilevers (OMCL-AC160TS, Olympus, Tokyo, Japan) with a typical resonant frequency of ∼300 kHz and a nominal tip radius of ∼7 nm. To evaluate the aminophosphonate layer thickness, we performed nanoshaving experiments by scanning a selected area in hard contact mode to selectively displace molecules and obtain an exposed substrate region. Shaving experiments were performed using Si_3_N_4_ cantilevers (OTESPA-R3, Bruker) with an elastic constant 26 N/m. Typical forces applied for shaving were in the range (200 ÷ 400) nN. After shaving, images with larger scan size were acquired in tapping mode. Data were analyzed with Gwyddion (v2.55) and JPKSPM Data Processing software (v7.0.162).

#### 2.2.2. X-ray Photoemission Spectroscopy

XPS measurements were performed using a PHI 5600 Multi-Technique apparatus (55317 Chanhassen, MN, USA) equipped with an X-ray Al-monochromatized source (hν 1486.6 eV), as previously reported [34,35]. The chamber is equipped with a neutralizer (low energy electron flood gun), used to avoid sample charging [36]. Survey spectra were acquired using a pass energy of 187.85 eV, while high resolution spectra were acquired with a pass energy of 23.50 eV. The binding energy scale was calibrated by setting the C1s component of adventitious carbon at a binding energy of 284.8 eV. A photoelectron take-off angle of 45° was chosen for data acquisition. To investigate the molecular orientation of aminophosphonates on flat TiO_2_ substrates, we performed angle-resolved XPS measurements with additional acquisitions at 20° and 70° take-off angles. Spectra were analyzed with CasaXPS processing software (v2.3.25PR1.0, Casa Software Ltd., Teignmouth, UK). Before spectra deconvolution, a Shirley background was subtracted from raw data. Voigt functions (30% Gaussian) were used for signal deconvolutions. For the P2p doublet deconvolution, a 0.86 eV spin-orbit splitting value was used with an area ratio between the main and the secondary component fixed at 2:1.

## 3. Results

### 3.1. Porous Titanium Oxide

**Figure 1:**
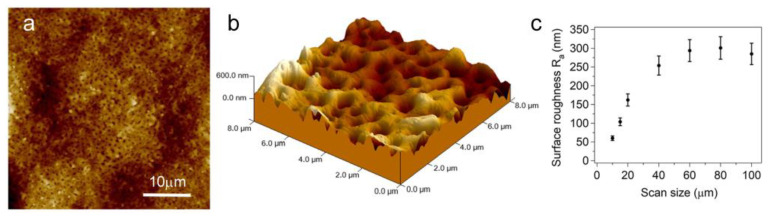
Typical AFM images of titanium anodized in a 1 H_2_SO_4_ aqueous solution with a limiting potential of 200 V: (**a**) top view image (z scale 1.5 µm). (**b**) Three-dimensional representation of the sample surface. (**c**) Surface roughness Ra vs. scan size.

Figure 1 shows a typical AFM image of a titanium sample anodized in 1 M H_2_SO_4_ aqueous solution with a limiting potential of 200 V (as additional information, a representative SEM image is reported in Figure S2). Anodization occurs in the anodic spark deposition regime as can be inferred from the presence of pores formed when the applied potential overcomes the oxide breakdown potential [37]. Oxide growth in the anodic spark deposition regime was chosen since a highly porous surface is known to favor implant osseointegration [38]. The porous structure observed in the large-scale top view image of Figure 1a can be better appreciated in the larger magnification 3D representation of the surface shown in Figure 1b. From the analysis of AFM images both surface roughness and pore size can be evaluated. Figure 1c shows a plot of the surface roughness as a function of the image size: surface roughness levels off asymptotically at values around 300 nm.

From the analysis of the z-profiles of the AFM images we can estimate pore diameter and depth. Pore diameter values are in the range (500 ÷ 800) nm while pore depths vary from 150 nm to 450 nm.

As reported in literature, changing the anodizing conditions (electrolyte, potential, current, time) allows one to modulate the oxide layer structure and composition in terms of pore size, oxide thickness and electrolyte inclusions [39,40,41,42,43]. Here, we chose 200 V anodizing in H_2_SO_4_ aqueous solution as an example of anodizing conditions that lead to the growth of a porous oxide layer suitable for biomaterial development.

Our goal is to investigate the feasibility of amino acid coupling to porous titanium oxide through an aminophosphonate linker as a first step to anchor short peptides, namely antimicrobial peptides, to the oxide surface.

The high inherent roughness of porous oxide makes it difficult to analyze the molecular structure of ultrathin films despite the use of highly sensitive methods such as AFM or XPS. We therefore resorted to parallel experiments on the self-assembly of aminophosphonates on TiO_2_ flat films to obtain details on deposition and molecular ordering on the surface that could guide the functionalization of porous titanium oxide.

### 3.2. Phosphonate Deposition on TiO*_2_* Surfaces

**Figure 2:**
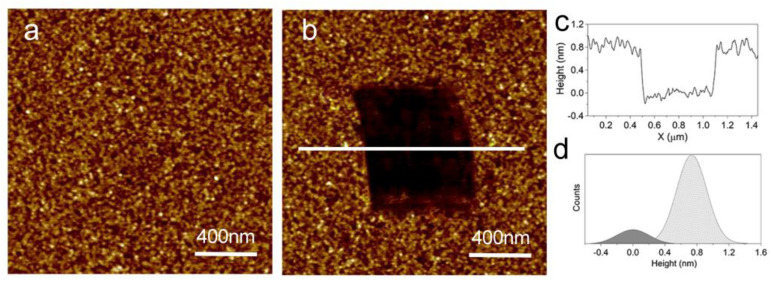
(**a**) Tapping mode AFM image of a sputtered TiO_2_ film (z-scale: 3 nm). (**b**) Tapping mode AFM image acquired after a nano-shaving experiment on aminophosphonate layer deposited on a sputtered TiO_2_ film (z-scale: 3 nm). (**c**) AFM z-profile along the white line shown in (**b**). (**d**) Height histogram of the image in (**b**).

To check for the formation of an aminophosphonate self-assembled monolayer on TiO_2_ flat substrates, we carried out AFM nanoshaving experiments. Figure 2a shows a typical AFM image of a flat TiO_2_ substrate. TiO_2_ substrates exhibit a polycrystalline structure with a surface roughness of 0.4 nm (over a 10 µm × 10 µm image). The effect of molecular deposition is hardly detectable from the analysis of the changes in sample morphology following molecular deposition. Conversely, AFM nanoshaving was successfully employed to evaluate the film thickness. In a nanoshaving experiment, molecules are selectively removed from a defined region of the sample by applying a high tip load [44,45]. In the present work molecule removal was obtained by tip scanning in hard contact mode. Figure 2b shows the result of a nanoshaving experiment on an aminophosphonate layer self-assembled on the TiO_2_ substrate. Soft tapping mode images acquired after removing the molecules show a dark area where the substrate is exposed. A direct evaluation of the molecular layer thickness can be inferred from the z-profiles of the images (an example is shown in Figure 2c) as the height difference between the molecule-covered region and the bare substrate. However, for a statistically significant evaluation of the layer thickness we can analyze the height histogram of the AFM images (an example is shown in Figure 2d). The histogram is characterized by two peaks. The oxide surface and the film correspond to the peaks centered at 0 nm and at ∼0.8 nm, respectively. The distance between the two peaks allows to evaluate the thickness of the aminophosphonate layer. A statistical analysis of different patches on several samples provided an average value of the film thickness of (0.8 ± 0.2) nm. This result points to the formation of an aminophosphonate monolayer with the molecular axis almost perpendicular to the surface. This result is in agreement with previous data obtained using polished titanium sheets as substrates [33] and makes us confident in using the same deposition method also on porous substrates.

As discussed in the following, further evidence of the formation of an oriented aminophosphonate monolayer was obtained by angle-resolved XPS analysis.

**Figure 3:**
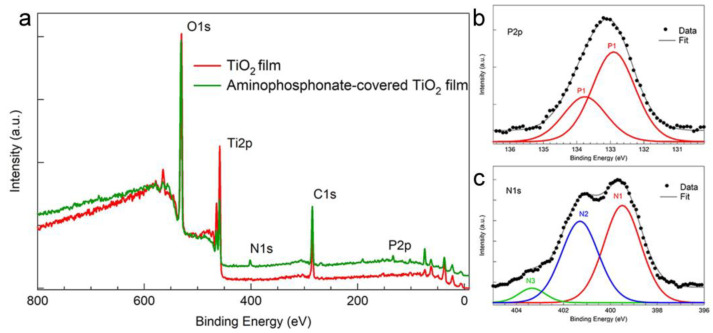
(**a**) XPS survey spectra of a TiO_2_ flat film before (red curve) and after (green curve) aminophosphonate deposition. (**b**,**c**) High resolution XPS spectra of P2p (**b**) and N1s (**c**) core level regions measured after aminophosphonate deposition on TiO_2_.

Wide scan XPS spectra were first acquired to investigate the surface compositional changes of the TiO_2_ substrate following molecular deposition. Figure 3a shows the survey spectra of the TiO_2_ substrate before (red line) and after (green line) aminophosphonate deposition.

The XPS survey spectrum of the substrate indicates the presence of titanium and oxygen with some adventitious carbon. In passing we note that very similar survey spectra characterize the porous TiO_2_ surface (see SI), with some additional sulphur traces (less than 1%) due to the anodization process. After the aminophosphonate deposition, phosphorous and nitrogen signals can be observed in the survey spectrum, thus confirming the molecular deposition. Substrate signals are easily detected in the spectrum due to the thinness of the deposited layer.

To investigate molecular binding to the substrate in more detail, we acquired high-resolution XPS spectra. The P2p core level region (Figure 3b) can be deconvoluted with a doublet, P1, each component with FWHM = 1.5 eV, with the 2p3/2 component at a binding energy of (132.9 ± 0.2) eV. The position of the P2p component is slightly shifted respect to the P2p component of the aminophosphonate powder [33] confirming that the binding state of the phosphonate has changed (from unbound to bound) [46].

The N1s core level region spectrum (Figure 3c) can be deconvoluted with three components with a FWHM of 1.8 eV and a binding energy of (399.6 ± 0.2) eV (N1), (401.3 ± 0.2) eV (N2) and (403.1 ± 0.2) eV (N3). The two main components, N1 and N2, can be attributed to the amine group, in its neutral (−NH_2_) and protonated state (−NH_3_^+^), respectively [47,48,49]. We note that the N1s signal of the aminophosphonate powder is characterized by only one component, N2, as expected since the starting compound is aminophosphonate chloride salt [33]. The coexistence of N1 and N2 components in the N1s signal of the aminophosphonate monolayers derives from the deprotonation of a fraction of −NH_3_^+^ groups in the monolayer due to electrostatic repulsion, as already reported for other self-assembled monolayers bearing ionizable groups [50].

The third minor component, N3, derives from the interaction of adventitious nitrogen with oxygen during plasma treatment, before the aminophosphonate deposition [51] and is observed also on plasma treated bare TiO_2_ substrates.

Due to the high flatness of the substrate, we could perform angle-resolved XPS measurements to investigate the molecular orientation of the aminophosphonate on the TiO_2_ surface.

The nitrogen and phosphorous signal intensities were evaluated from the deconvolution of the high-resolution spectra of the N1s and P2p core level regions acquired at 20°, 45° and 70° take-off angles. The N/P intensity ratios at the different take-off angles are reported in Table 1.

Increasing the detector take-off angle results in a decrease in the N/P ratio, indicating that the nitrogen atoms are localized above the phosphorous ones. Considering the aminophosphonate structure, the N/P dependence on the take-off angle confirms the binding of the molecule to the substrate via the phosphonate head and the exposure of the amino group towards the outer monolayer interface. As discussed in the following, the availability of exposed amino groups can be exploited for the further coupling with amino acids.

In the case of aminophosphonate deposition on porous titanium oxide, the high surface roughness of the substrate hinders a meaningful analysis of angle-resolved XPS measurements. Nonetheless, the high similarity between the P2p and N1s high-resolution spectra acquired after aminophosphonate deposition on flat and porous TiO_2_ substrates makes us confident in assuming the same molecular organization on both substrates. Indeed, the formation of a monolayer that exposes amino groups also on porous substrates is confirmed by the amino acid coupling experiments discussed below.

### 3.3. Leucine Coupling

**Figure 4:**
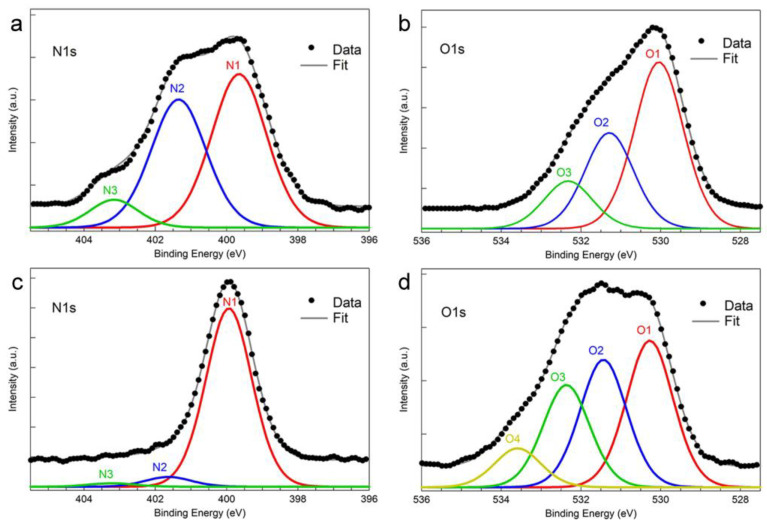
(**a**,**c**) N1s and (**b**,**d**) O1s high-resolution spectra acquired on aminophosphonate monolayers self-assembled on porous TiO_2_ before (**a**,**b**) and after (**c**,**d**) Fmoc-Leucine coupling.

Since Fmoc-Leucine contains both nitrogen and oxygen atoms, these two elements were chosen as reference signals to investigate Fmoc-Leucine coupling to aminophosphonate functionalized TiO_2_ porous substrates. To highlight the changes in the N1s and O1s spectra introduced by the amino acid deposition, in Figure 4 we report the high-resolution spectra of the N1s and O1s core lever regions acquired before (Figure 4a,b) and after (Figure 4c,d) Fmoc-Leucine coupling.

The N1s spectrum of the aminophosphonate-covered porous substrate (Figure 4a) can be deconvoluted with three components with a FWHM of 1.8 eV. The binding energy of N1, N2 and N3 components are (399.5 ± 0.2) eV, (401.3 ± 0.2) eV and (403.3 ± 0.2) eV, respectively. It is worth to note that the binding energy positions of N1, N2, and N3 obtained on aminophosphonate monolayers deposited on porous TiO_2_ are consistent, within the experimental uncertainties, with those obtained on aminophosphonate monolayers deposited on flat TiO_2_ substrates.

A significant change is observed in the N1s line-shape after Fmoc-Leucine coupling. The N1s spectrum (Figure 4c) now shows a dominant component N1 at a binding energy of (399.9 ± 0.2) eV assignable to the N−C=O of the peptide bond between aminophosphonate and Leucine and between Leucine and Fmoc [52,53]. The position of the N2 and N3 components, at binding energies of (401.6 ± 0.2) eV and of (402.9 ± 0.2) eV, respectively, are consistent, within the experimental variability, with the positions and attributions reported above for the aminophosphonate monolayers. The low intensity of N2 compared to N1 suggests a high coupling yield between Fmoc-Leucine and aminophosphonate, with a low content of residual unbound −NH_3_^+^ groups. Moreover, the deposition of Fmoc-Leucine on top of aminophosphonate partially screens the N2 and N3 component reducing their intensity.

The O1s region of the aminophosphonate-covered porous substrate reported in Figure 4b can be deconvoluted with three components, each with a FWHM of 1.4 eV. The O1 component at a binding energy of (530.0 ± 0.2) eV derives from the TiO_2_ substrate [54,55]. The O2 component at a binding energy of (531.3 ± 0.2) eV can be assigned to the Ti−O−P bond, confirming the binding of the aminophosphonate to the substrate [33,56]. The last component O3 at a binding energy of (532.3 ± 0.2) eV can be mainly assigned to phosphonate P=O [55,57].

The Fmoc-Leucine coupling to the aminophosphonate monolayer induces significant changes also in the O1s signal (Figure 4d). After Fmoc-Leucine coupling, the deconvolution of the O1s spectrum needs four components, all with a FWHM of 1.4 eV. The O1 component at (530.2 ± 0.2) eV and the O2 component at (531.4 ± 0.2) eV are assigned to TiO_2_ and to Ti−O−P binding [56], respectively, similarly to the O1s analysis before Fmoc-Leucine coupling. The third component, O3, at a binding energy of (532.4 ± 0.2) eV is assigned to the oxygen O=C−N involved in the peptide bond both between Leucine and aminophosphonate and between Fmoc and Leucine [53,58]. As reported above, some P=O of the phosphonate group can contribute to O3 as well. The last component, O4, at a binding energy of (533.6 ± 0.2) eV is assignable to the aliphatic oxygen in the O−(C=O) −C bond of Fmoc [58] (see SI for Fmoc-Leucine structural formula). We note that the intensity of O3 is roughly twice that of O4. This is in reasonable agreement with the fact that a 2:1 ratio of oxygen engaged in peptide bond (O3) to aliphatic oxygen (O4) is expected, as a result of bond formation between Fmoc-Leucine and aminophosphonate.

The comparison of the O1s region before (Figure 4b) and after (Figure 4d) the coupling with Fmoc-Leucine shows a significant reduction in the O1 intensity compared the other components. This finding confirms the deposition of Fmoc-Leucine which further hinders the detection of photoelectrons emitted from deeper regions, i.e., electrons photoemitted from the TiO_2_ substrate.

## 4. Conclusions

We studied the coupling of an amino acid, leucine, to the surface of porous titanium oxide obtained by anodic oxidation. We followed a two-step deposition process: self-assembly of aminophosphonates followed by the formation of a peptide bond between the amino acid and the aminophosphonate. Fmoc was used to protect the amine group of Leucine during the deposition process. To ensure efficient coupling between aminophosphonates and amino acids, an oriented aminophosphonate monolayer exposing the amino group must be formed. To verify the proper orientation of aminophosphonates on the surface, we performed parallel experiments of aminophosphonate deposition on flat titanium oxide. By AFM nanoshaving and angle-resolved XPS measurements on flat substrates, we could verify the formation of an oriented aminophosphonate monolayer exposing the amine group. Supported by the high consistency of XPS results obtained on aminophosphonate monolayers deposited on flat and porous oxide, we applied the same functionalization procedure on anodized titanium to obtain porous surfaces with exposed amine groups. Indeed, the functionalization approach, applied to porous titanium oxide, enabled the coupling of Fmoc-Leucine to the surface as revealed by high resolution XPS analysis.

The method, used here for leucine, can be extended to other amino acids and peptides. From the perspective of biomaterials design, the development of antimicrobial coatings that can reduce the risk of periprosthetic infections is of particular interest. Under this perspective, further studies will focus on anchoring antimicrobial peptides to porous titanium oxide to impart antimicrobial properties to the implant surface.

## Figures and Tables

**Table 1:** N/P intensity ratio as a function of the take-off angle.

Take-Off Angle	N/P
20°	1.2
45°	1.1
70°	0.8

## Data Availability

The data presented in this study are available on request from the corresponding author.

## Supplementary Materials

The following supporting information can be downloaded at: https://www.mdpi.com/article/10.3390/ma16020784/s1, Figure S1: structural formula of Fmoc-Leucine-OH; Figure S2: SEM image of titanium foil anodized in 1 M H_2_SO_4_ aqueous solution at 200 V limiting potential; Figure S3: survey spectrum of titanium foil anodized in 1 M H_2_SO_4_ aqueous solution at 200 V limiting potential.

## References

[1] Brudzisz A.M., Giziński D., Stępniowski W.J. (2021). Incorporation of Ions into Nanostructured Anodic Oxides—Mechanism and Functionalities. Molecules.

[2] Casanova L., Vicentini L., Pedeferri M., Ormellese M. (2021). Unipolar Plasma Electrolytic Oxidation: Waveform Optimisation for Corrosion Resistance of Commercially Pure Titanium. Mater. Corros..

[3] Caetano G.L., dos Santos Junior J., Pereira B.L., Benegra M. (2021). One-Step Plasma Electrolytic Oxidation in Ti-30Nb-8Zr, Ti, and Nb Surfaces. Surf. Eng..

[4] Canepa P., Firpo G., Mattera L., Canepa M., Cavalleri O. (2020). Calcium and Phosphorous Enrichment of Porous Niobium and Titanium Oxides for Biomaterial Applications. Surf. Coat. Technol..

[5] Quintero D., Gómez M.A., Araujo W.S., Echeverría F., Calderón J.A. (2019). Influence of the Electrical Parameters of the Anodizing PEO Process on Wear and Corrosion Resistance of Niobium. Surf. Coat. Technol..

[6] Pereira B.L., da Luz A.R., Lepienski C.M., Mazzaro I., Kuromoto N.K. (2018). Niobium Treated by Plasma Electrolytic Oxidation with Calcium and Phosphorus Electrolytes. J. Mech. Behav. Biomed. Mater..

[7] Chen Z.-Y., Gao S., Zhang Y.-W., Zhou R.-B., Zhou F. (2021). Antibacterial Biomaterials in Bone Tissue Engineering. J. Mater. Chem. B.

[8] Busscher H.J., van der Mei H.C., Subbiahdoss G., Jutte P.C., van den Dungen J.J.A.M., Zaat S.A.J., Schultz M.J., Grainger D.W. (2012). Biomaterial-Associated Infection: Locating the Finish Line in the Race for the Surface. Sci. Transl. Med..

[9] Ghimire A., Song J. (2021). Anti-Periprosthetic Infection Strategies: From Implant Surface Topographical Engineering to Smart Drug-Releasing Coatings. ACS Appl. Mater. Interfaces.

[10] Linklater D.P., Baulin V.A., Juodkazis S., Crawford R.J., Stoodley P., Ivanova E.P. (2021). Mechano-Bactericidal Actions of Nanostructured Surfaces. Nat. Rev. Microbiol..

[11] Cao Y., Su B., Chinnaraj S., Jana S., Bowen L., Charlton S., Duan P., Jakubovics N.S., Chen J. (2018). Nanostructured Titanium Surfaces Exhibit Recalcitrance towards Staphylococcus Epidermidis Biofilm Formation. Sci. Rep..

[12] Ganjian M., Modaresifar K., Zhang H., Hagedoorn P.-L., Fratila-Apachitei L.E., Zadpoor A.A. (2019). Reactive Ion Etching for Fabrication of Biofunctional Titanium Nanostructures. Sci. Rep..

[13] Buxadera-Palomero J., Albó K., Gil F.J., Mas-Moruno C., Rodríguez D. (2020). Polyethylene Glycol Pulsed Electrodeposition for the Development of Antifouling Coatings on Titanium. Coatings.

[14] Solano I., Parisse P., Gramazio F., Cavalleri O., Bracco G., Castronovo M., Casalis L., Canepa M. (2015). Spectroscopic Ellipsometry Meets AFM Nanolithography: About Hydration of Bio-Inert Oligo(Ethylene Glycol)-Terminated Self Assembled Monolayers on Gold. Phys. Chem. Chem. Phys..

[15] Es-Souni M., Es-Souni M., Bakhti H., Gülses A., Fischer-Brandies H., Açil Y., Wiltfang J., Flörke C. (2021). A Bacteria and Cell Repellent Zwitterionic Polymer Coating on Titanium Base Substrates towards Smart Implant Devices. Polymers.

[16] Zhang B., Skelly J.D., Braun B.M., Ayers D.C., Song J. (2020). Surface-Grafted Zwitterionic Polymers Improve the Efficacy of a Single Antibiotic Injection in Suppressing *Staphylococcus Aureus* Periprosthetic Infections. ACS Appl. Bio. Mater..

[17] Celesti C., Gervasi T., Cicero N., Giofrè S.V., Espro C., Piperopoulos E., Gabriele B., Mancuso R., Lo Vecchio G., Iannazzo D. (2022). Titanium Surface Modification for Implantable Medical Devices with Anti-Bacterial Adhesion Properties. Materials.

[18] Villegas M., Zhang Y., Badv M., Alonso-Cantu C., Wilson D., Hosseinidoust Z., Didar T.F. (2022). Enhancing Osseointegration and Mitigating Bacterial Biofilms on Medical-Grade Titanium with Chitosan-Conjugated Liquid-Infused Coatings. Sci. Rep..

[19] Slate A.J., Wickens D.J., El Mohtadi M., Dempsey-Hibbert N., West G., Banks C.E., Whitehead K.A. (2018). Antimicrobial Activity of Ti-ZrN/Ag Coatings for Use in Biomaterial Applications. Sci. Rep..

[20] Huan Y., Kong Q., Mou H., Yi H. (2020). Antimicrobial Peptides: Classification, Design, Application and Research Progress in Multiple Fields. Front. Microbiol..

[21] Diefenbeck M., Schrader C., Gras F., Mückley T., Schmidt J., Zankovych S., Bossert J., Jandt K.D., Völpel A., Sigusch B.W. (2016). Gentamicin Coating of Plasma Chemical Oxidized Titanium Alloy Prevents Implant-Related Osteomyelitis in Rats. Biomaterials.

[22] Wang S., Song J., Li Y., Zhao X., Chen L., Li G., Wang L., Jia Z., Ge X. (2019). Grafting Antibacterial Polymer Brushes from Titanium Surface via Polydopamine Chemistry and Activators Regenerated by Electron Transfer ATRP. React. Funct. Polym..

[23] Sánchez-Bodón J., Andrade del Olmo J., Alonso J.M., Moreno-Benítez I., Vilas-Vilela J.L., Pérez-Álvarez L. (2021). Bioactive Coatings on Titanium: A Review on Hydroxylation, Self-Assembled Monolayers (SAMs) and Surface Modification Strategies. Polymers.

[24] Lee I. (2013). Molecular Self-Assembly: Smart Design of Surface and Interface via Secondary Molecular Interactions. Langmuir.

[25] Pujari S.P., Scheres L., Marcelis A.T.M., Zuilhof H. (2014). Covalent Surface Modification of Oxide Surfaces. Angew. Chem. Int. Ed..

[26] Bordi F., Prato M., Cavalleri O., Cametti C., Canepa M., Gliozzi A. (2004). Azurin Self-Assembled Monolayers Characterized by Coupling Electrical Impedance Spectroscopy and Spectroscopic Ellipsometry. J. Phys. Chem. B.

[27] Dubey M., Weidner T., Gamble L.J., Castner D.G. (2010). Structure and Order of Phosphonic Acid-Based Self-Assembled Monolayers on Si(100). Langmuir.

[28] Cattani-Scholz A. (2017). Functional Organophosphonate Interfaces for Nanotechnology: A Review. ACS Appl. Mater. Interfaces.

[29] Shannon F.J., Cottrell J.M., Deng X.-H., Crowder K.N., Doty S.B., Avaltroni M.J., Warren R.F., Wright T.M., Schwartz J. (2008). A Novel Surface Treatment for Porous Metallic Implants That Improves the Rate of Bony Ongrowth. J. Biomed. Mater. Res..

[30] Bozzini S., Petrini P., Tanzi M.C., Zürcher S., Tosatti S. (2010). Poly(Ethylene Glycol) and Hydroxy Functionalized Alkane Phosphate Mixed Self-Assembled Monolayers to Control Nonspecific Adsorption of Proteins on Titanium Oxide Surfaces. Langmuir.

[31] Danahy M.P., Avaltroni M.J., Midwood K.S., Schwarzbauer J.E., Schwartz J. (2004). Self-Assembled Monolayers of α,ω-Diphosphonic Acids on Ti Enable Complete or Spatially Controlled Surface Derivatization. Langmuir.

[32] Zhu L., Gregurec D., Reviakine I. (2013). Nanoscale Departures: Excess Lipid Leaving the Surface during Supported Lipid Bilayer Formation. Langmuir.

[33] Canepa P., Gonella G., Pinto G., Grachev V., Canepa M., Cavalleri O. (2019). Anchoring of Aminophosphonates on Titanium Oxide for Biomolecular Coupling. J. Phys. Chem. C.

[34] Toccafondi C., Uttiya S., Cavalleri O., Gemme G., Barborini E., Bisio F., Canepa M. (2014). Optical Properties of Nanogranular and Highly Porous TiO_2_ Thin Films. J. Phys. D Appl. Phys..

[35] Canepa P., Solano I., Uttiya S., Gemme G., Rolandi R., Canepa M., Cavalleri O. (2017). Phosphonate Molecular Layers on TiO_2_ Surfaces. MATEC Web Conf..

[36] Blangiardo A., Lagomarsino G., Basso A., Canepa P., Cavalleri O., Rossi S., Monticelli O. (2021). Preparation, Application and Recycling of a Catalytic Microflow Reactor Based on Polylactic Acid. Appl. Surf. Sci..

[37] Afshar A., Vaezi M.R. (2004). Evaluation of Electrical Breakdown of Anodic Films on Titanium in Phosphate-Base Solutions. Surf. Coat. Technol..

[38] Martinez M.A.F., Balderrama Í.d.F., Karam P.S.B.H., de Oliveira R.C., de Oliveira F.A., Grandini C.R., Vicente F.B., Stavropoulos A., Zangrando M.S.R., Sant’Ana A.C.P. (2020). Surface Roughness of Titanium Disks Influences the Adhesion, Proliferation and Differentiation of Osteogenic Properties Derived from Human. Int. J. Implant Dent..

[39] Michalska J., Sowa M., Piotrowska M., Widziołek M., Tylko G., Dercz G., Socha R.P., Osyczka A.M., Simka W. (2019). Incorporation of Ca Ions into Anodic Oxide Coatings on the Ti-13Nb-13Zr Alloy by Plasma Electrolytic Oxidation. Mater. Sci. Eng. C.

[40] Stępniowski W.J., Nowak-Stępniowska A., Presz A., Czujko T., Varin R.A. (2014). The Effects of Time and Temperature on the Arrangement of Anodic Aluminum Oxide Nanopores. Mater. Charact..

[41] Canepa P., Ghiara G., Spotorno R., Canepa M., Cavalleri O. (2021). Structural vs. Electrochemical Investigation of Niobium Oxide Layers Anodically Grown in a Ca and P Containing Electrolyte. J. Alloy. Compd..

[42] Canepa P., Firpo G., Gatta E., Spotorno R., Giannoni P., Quarto R., Canepa M., Cavalleri O. (2022). A Two-Step Approach to Tune the Micro and Nanoscale Morphology of Porous Niobium Oxide to Promote Osteointegration. Materials.

[43] Pereira B.L., Lepienski C.M., Seba V., Nugent M.J.D., Torres R., Kuroda P.A.B., Grandini C.R., Soares P. (2022). Plasma Electrolytic Oxidation up to Four-Steps Performed on Niobium and Nb-Ti Alloys. Surf. Coat. Technol..

[44] Pinto G., Canepa P., Canale C., Canepa M., Cavalleri O. (2020). Morphological and Mechanical Characterization of DNA SAMs Combining Nanolithography with AFM and Optical Methods. Materials.

[45] Pinto G., Parisse P., Solano I., Canepa P., Canepa M., Casalis L., Cavalleri O. (2019). Functionalizing Gold with Single Strand DNA: Novel Insight into Optical Properties via Combined Spectroscopic Ellipsometry and Nanolithography Measurements. Soft Matter.

[46] Luschtinetz R., Frenzel J., Milek T., Seifert G. (2009). Adsorption of Phosphonic Acid at the TiO_2_ Anatase (101) and Rutile (110) Surfaces. J. Phys. Chem. C.

[47] Caprile L., Cossaro A., Falletta E., Della Pina C., Cavalleri O., Rolandi R., Terreni S., Ferrando R., Rossi M., Floreano L. (2012). Interaction of L-Cysteine with Naked Gold Nanoparticles Supported on HOPG: A High Resolution XPS Investigation. Nanoscale.

[48] Cavalleri O., Gonella G., Terreni S., Vignolo M., Floreano L., Morgante A., Canepa M., Rolandi R. (2004). High Resolution X-Ray Photoelectron Spectroscopy of l-Cysteine Self-Assembled Films. Phys. Chem. Chem. Phys..

[49] Uvdal K., Bodö P., Liedberg B. (1992). L-Cysteine Adsorbed on Gold and Copper: An X-Ray Photoelectron Spectroscopy Study. J. Colloid Interface Sci..

[50] Fears K.P., Creager S.E., Latour R.A. (2008). Determination of the Surface p *K* of Carboxylic- and Amine-Terminated Alkanethiols Using Surface Plasmon Resonance Spectroscopy. Langmuir.

[51] Pashutski A., Folman M. (1989). Low temperature XPS studies of NO and N_2_O adsorption on Al(100). Surf. Sci..

[52] Lhoest J.-B., Bartiaux S., Gerin P.A., Genet M.J., Bertrand P., Rouxhet P.G. (2021). Poly(Amino Acids) by XPS: Analysis of Poly-L-Leucine. Surf. Sci. Spectra.

[53] Stevens J.S., de Luca A.C., Pelendritis M., Terenghi G., Downes S., Schroeder S.L.M. (2013). Quantitative Analysis of Complex Amino Acids and RGD Peptides by X-Ray Photoelectron Spectroscopy (XPS). Surf. Interface Anal..

[54] Tosatti S., Michel R., Textor M., Spencer N.D. (2002). Self-Assembled Monolayers of Dodecyl and Hydroxy-Dodecyl Phosphates on Both Smooth and Rough Titanium and Titanium Oxide Surfaces. Langmuir.

[55] Wagstaffe M., Thomas A.G., Jackman M.J., Torres-Molina M., Syres K.L., Handrup K. (2016). An Experimental Investigation of the Adsorption of a Phosphonic Acid on the Anatase TiO_2_ (101) Surface. J. Phys. Chem. C.

[56] Adolphi B., Jähne E., Busch G., Cai X. (2004). Characterization of the Adsorption of ω-(Thiophene-3-Yl Alkyl) Phosphonic Acid on Metal Oxides with AR-XPS. Anal. Bioanal. Chem..

[57] Tsud N., Yoshitake M. (2007). Vacuum Vapour Deposition of Phenylphosphonic Acid on Amorphous Alumina. Surf. Sci..

[58] Beamson G., Briggs D. (1993). High Resolution XPS of Organic Polymers: The Scienta ESCA300 Database. J. Chem. Educ..

