# Supplementary material for: Biofunctionalization of Porous Titanium Oxide through Amino Acid Coupling for Biomaterial Design
Source: Materials (Basel). 2023 Jan 13;16(2):784. doi: 10.3390/ma16020784 (PMC9865921; doi:10.3390/ma16020784)
Supplement: Supplementary file 1 [file materials-16-00784-s001.zip › materials-2165406-supplementary.pdf]

materials

Support Information

Biofunctionalization of Porous Titanium Oxide through Amino Acid Coupling for Biomaterial Design

Paolo Canepa [1], Danijela Gregurec [2], Nara Liessi [3], Silvia Maria Cristina Rotondi [1], Sergio Enrique Moya [4], Enrico Millo [3], Maurizio Canepa [1,5] and Ornella Cavalleri [1,*]

[1] Dipartimento di Fisica and OPTMATLAB, Università di Genova, Via Dodecaneso 33, 16146 Genova, Italy
[2] Department of Chemistry and Pharmacy, Chair of Aroma and Smell Research, Friedrich-Alexander-Universität Erlangen-Nürnberg, Schlossplatz 4, 91054 Erlangen, Germany
[3] Dipartimento di Medicina Sperimentale, Università di Genova, Viale Benedetto XV 1, 16132 Genova, Italy
[4] Soft Matter Nanotechnology Group, CIC biomaGUNE, Basque Research and Technology Alliance (BRTA), Paseo Miramón 182, 20014 San Sebastián, Spain
[5] INFN, Sezione di Genova, Via Dodecaneso 33, 16146 Genova, Italy
* Correspondence: cavalleri@fisica.unige.it

Keywords: porous titanium oxide; aminophosphonates; amino acid coupling; Fmoc-Leucine; nanoshaving; AFM; XPS

S1 – Structural formula of Fmoc-Leucine-OH

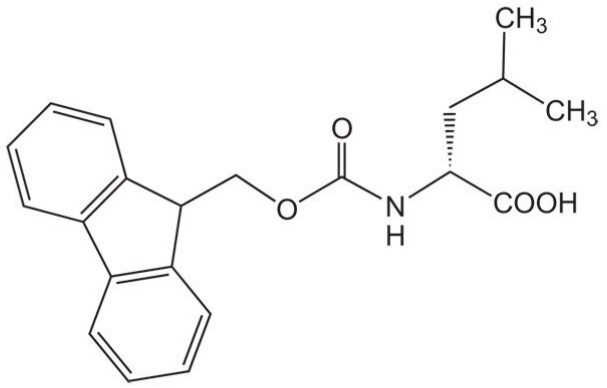

Figure S1. Structural formula of Fmoc-Leucine-OH.

S2- SEM analysis of anodized TiO_2 substrates

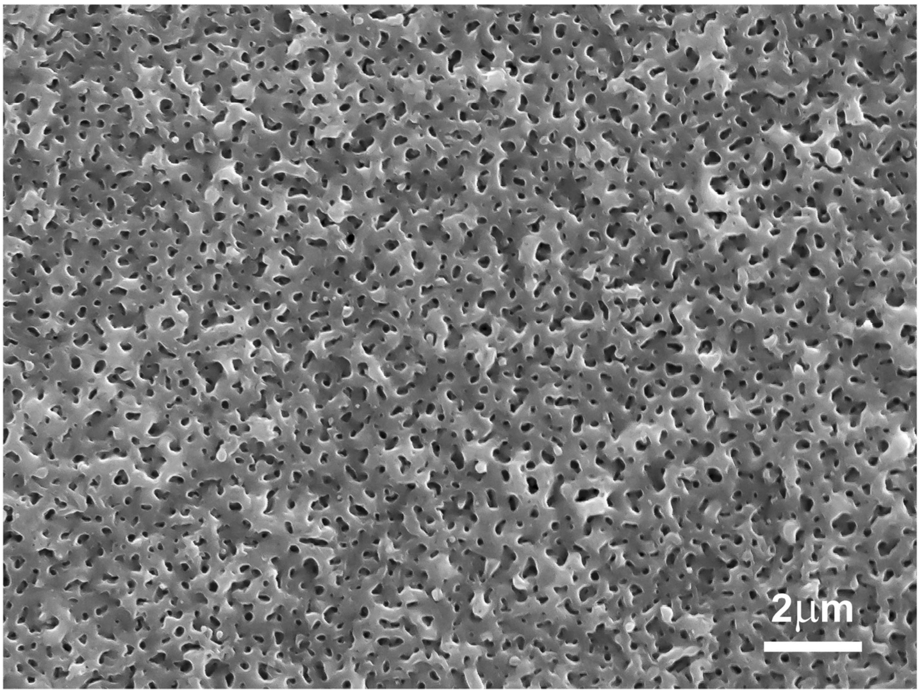

Figure S2. SEM image of titanium foil anodized in 1M H_2SO_4 aqueous solution at 200 V limiting potential.

Figure S2 shows a representative SEM image of anodized TiO_2 substrates. SEM measurements were performed using a LEO1450VP SEM with tungsten filament. Images were acquired using a secondary electron detector. The analysis was carried out in high vacuum conditions, without any metallization of the sample.

S3 – Porous TiO_2 compositional analysis

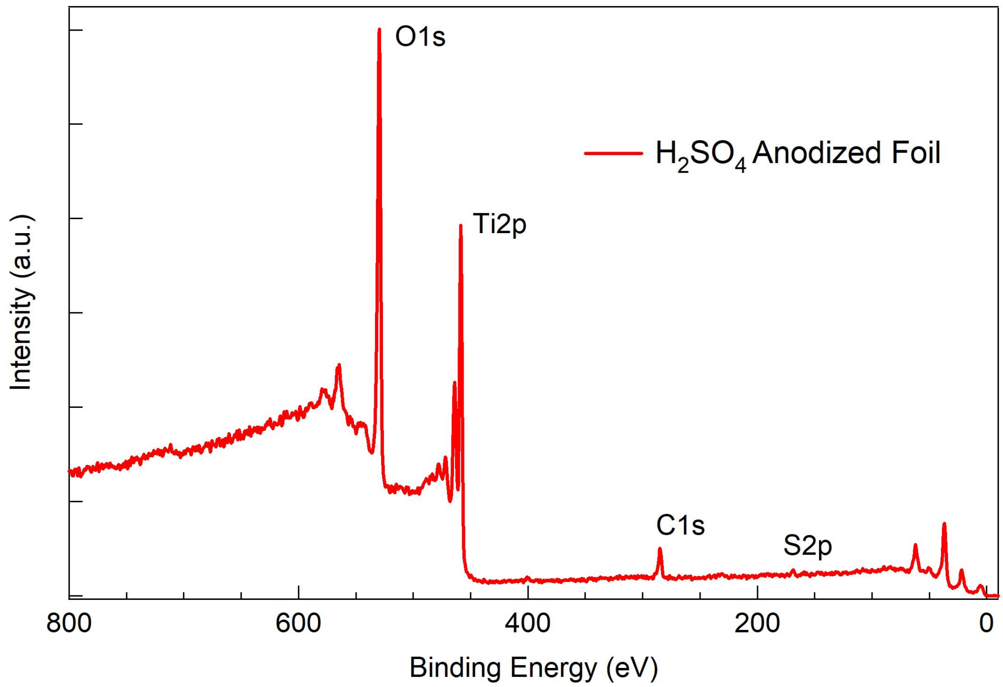

Figure S3. Survey spectrum of titanium foil anodized in 1M H_2SO_4 aqueous solution at 200 V limiting potential.

Figure S3 reports a typical XPS survey spectrum of a titanium foil anodized in a 1 M H_2SO_4 aqueous solution. As expected, the surface chemical composition shows mainly the presence of titanium (at a binding energy of about 458 eV) and oxygen (at a binding energy of about 530 eV). The tiny signal around 168 eV is assignable to sulphur, deriving from sulphur inclusions due to the used electrolyte. Moreover, some adventitious carbon (around 285 eV) is observable.
